# Evolution of Clinical Phenotypes of COVID-19 Patients During Intensive Care Treatment: An Unsupervised Machine Learning Analysis

**DOI:** 10.1177/08850666231153393

**Published:** 2023-02-06

**Authors:** Sander Siepel, Tariq A. Dam, Lucas M. Fleuren, Armand R.J. Girbes, Mark Hoogendoorn, Patrick J. Thoral, Paul W.G. Elbers, Frank C. Bennis, On behalf of the Dutch ICU Data Sharing Collaborators

**Affiliations:** 1 Quantitative Data Analytics Group, 318230 Department of Computer Science, Faculty of Science, 1190 Vrije Universiteit, Amsterdam, the Netherlands; 2 Department of Intensive Care Medicine, Laboratory for Critical Care Computational Intelligence, Amsterdam Medical Data Science, 26066 Amsterdam UMC, 1190 Vrije Universiteit, Amsterdam, the Netherlands; 3 Nivel, Netherlands Institute for Health Services Research, Utrecht, the Netherlands

**Keywords:** coronavirus disease 2019, intensive care, clustering, clinical phenotypes, endotypes, subphenotypes, clinical phenotype half-life

## Abstract

**Background:**

Identification of clinical phenotypes in critically ill COVID-19 patients could improve understanding of the disease heterogeneity and enable prognostic and predictive enrichment. However, previous attempts did not take into account temporal dynamics with high granularity. By including the dimension of time, we aim to gain further insights into the heterogeneity of COVID-19.

**Methods:**

We used granular data from 3202 adult COVID patients in the Dutch Data Warehouse that were admitted to one of 25 Dutch ICUs between February 2020 and March 2021. Parameters including demographics, clinical observations, medications, laboratory values, vital signs, and data from life support devices were selected. Twenty-one datasets were created that each covered 24 h of ICU data for each day of ICU treatment. Clinical phenotypes in each dataset were identified by performing cluster analyses. Both evolution of the clinical phenotypes over time and patient allocation to these clusters over time were tracked.

**Results:**

The final patient cohort consisted of 2438 COVID-19 patients with a ICU mortality outcome. Forty-one parameters were chosen for cluster analysis. On admission, both a mild and a severe clinical phenotype were found. After day 4, the severe phenotype split into an intermediate and a severe phenotype for 11 consecutive days. Heterogeneity between phenotypes appears to be driven by inflammation and dead space ventilation. During the 21-day period, only 8.2% and 4.6% of patients in the initial mild and severe clusters remained assigned to the same phenotype respectively. The clinical phenotype half-life was between 5 and 6 days for the mild and severe phenotypes, and about 3 days for the medium severe phenotype.

**Conclusions:**

Patients typically do not remain in the same cluster throughout intensive care treatment. This may have important implications for prognostic or predictive enrichment. Prominent dissimilarities between clinical phenotypes are predominantly driven by inflammation and dead space ventilation.

## Background

The world continues to suffer from the ongoing coronavirus disease 2019 (COVID-19) pandemic. COVID-19 is one of the deadliest pandemics in history with more than 360 million confirmed cases as of January 2022, and 5.6 million deaths.^1^ COVID-19 and measures to control it cause significant social and economic disruptions and put unprecedented pressure on healthcare systems worldwide.

Despite major scientific effort, many aspects of COVID-19 remain poorly understood. This includes the identification of distinct clinical phenotypes in critically ill COVID-19 patients. The identification of clinical phenotypes could improve the understanding of disease heterogeneity and the response to treatment. Furthermore, it may enable prognostic enrichment, facilitating better outcome estimation for patient selection in trials and for shared decision making, like limitations of treatment. Finally, it could also provide predictive enrichment, which is the selection of patients who are more likely to respond to a given therapy on the basis of a biological mechanism. Predictive enrichment could thus identify potential targets for more personalized COVID-19 treatment throughout the course of critical illness, as previously shown for sepsis.^2^

Previous attempts to identify clinical COVID-19 phenotypes using unsupervised machine learning techniques such as clustering analyses have revealed important signals of heterogeneity within the disease by identifying distinct clusters.^3–7^ However, these analyses only considered data that is available on admission and up to 48 h thereafter. This static approach ignores the dynamics of the disease and the response to therapy. Thus, it is currently unknown whether clinical phenotypes in COVID-19 patients are stable and whether or not this could impact predictive and prognostic enrichment.

The Dutch ICU Data Warehouse (DDW) contains millions of data points on thousands of critically ill COVID-19 patients for the full duration of their treatment at one of 25 intensive care units in the Netherlands.^8^ These data may be leveraged to gain new insights into the heterogeneity of COVID-19 by adding the dimension of time to the search for clinical phenotypes.

We hypothesized that this approach could reveal insights into the evolution of clinical phenotypes (ie how they change over time) and patient allocation to these clinical phenotypes over time. We therefore used the DDW to study this dynamic process, aiming to identify deterioration or recovery as well as potentially modifiable factors in critically ill COVID-19 patients throughout the course of their intensive care treatment.

## Methods

All data was retrieved from the Dutch ICU Data Warehouse, a large, multi-center and full admission electronic health record data dataset. The DDW contains high-granular data on 3202 critically ill COVID-19 patients admitted to one of 25 Dutch ICUs between February 2020 and March 2021. All data are pseudonymized. The institutional review board of Amsterdam UMC, location VUmc waived the need for informed consent from individual patients and approved of an opt-out procedure.

### Patients and Parameters

All patients in the DDW were eligible for inclusion if ICU mortality outcome data were available. Transferred patients were included if the transfer destination data were available. Relevant parameters were selected by a team of intensivists aiming to include a balanced representation of all organ systems. Among the clinically relevant parameters, only those for which at least 50% of the patients had a stored value for this parameter within the first 24 h of ICU admission were included. Determination of this threshold was done on the basis of the trade-off between the number of available parameters for selection, and the risk of introducing a significant bias in the dataset by imputation.

### Data Preprocessing

**Figure 1.**
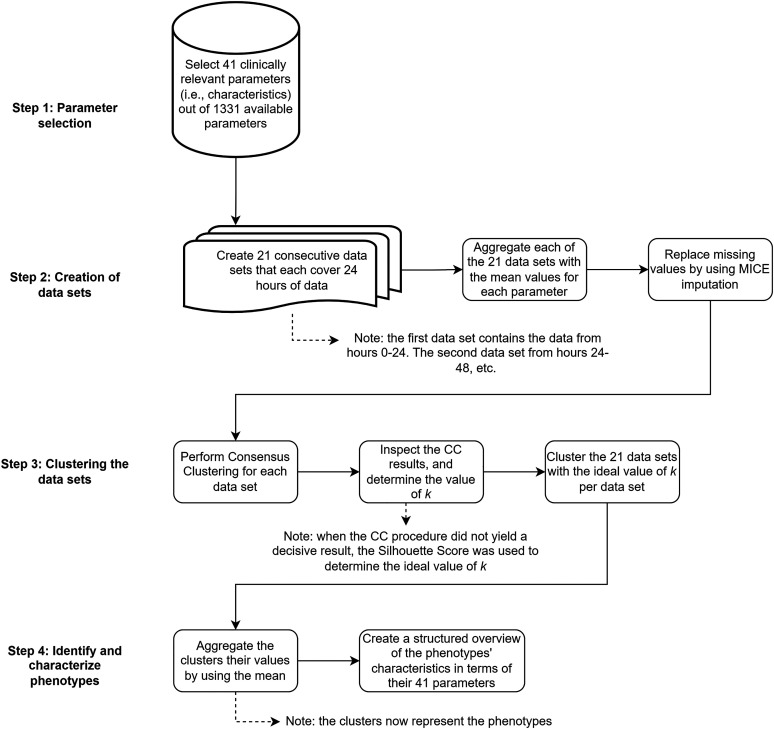
Flowchart of the methods used from collection up to analysis. Notice that the number of parameters is strongly reduced, that a data set is created and analyzed for each separate day, and that the number of clusters per day can differ.

The preprocessing and clustering procedure is visualized in Figure 1. All analyses were carried out in Python 3.8 (Python software foundation). To investigate the evolution of clinical phenotypes and patient allocation to those over time, multiple cluster analyses were performed for different time points during ICU treatment. To this end, consecutive data sets were created that each covered the data of the next 24 h, ie the first data set covered 0–24 h, the second data set 24–48 h, etc The selected number of days was set to 21, ensuring that a reasonable number of patients remained available for cluster analysis in the last data set (480-504 h after admission). Having a reasonable number of patients was necessary to ensure the validity of our results, as we performed several data aggregations, and too few patients result in unstable clustering. Therefore, the trade-off between time and patients was set at three weeks. Temporal data were aggregated as the mean or median value (as appropriate) for each patient and each parameter, for every data set. Medication data were transformed to binary parameters indicating whether the medication was administered during the 24-h period. For each parameter presenting with missing values, the Multiple Imputation by Chained Equations (MICE) procedure using the miceforest package was deployed to replace the missing data with substituted values. Default settings using lightgbm were followed and the amount of iterations was set to 15, based on the average percentage of missing values, as recommended by Bodner^9^ and White, Royston, and Wood.^10^

### Clustering

The k-medoids clustering algorithm with Partitioning Around Medoids (PAM)^11^ was used to cluster the datasets. Distance matrices as input for the clustering algorithm were created separately for each data set with the Gower's Distance metric.^12^ This metric was chosen because of its ability to account for both numeric and non-numeric data. To determine the amount of clusters k for each data set, Consensus Clustering (CC) was performed with 500 iterations on samples with 80% data retention for each value of k between 2 and 7, as recommended by Monti et al.^13^ These numbers are based on previous research, which has shown that similar patient cohorts are best divided into either two, three, or four clusters.^3–5^ Both color-coded heatmaps and cumulative distribution function (CDF) plots were used to visually identify the ideal amount of clusters k for each of the 21 data sets, ie the number of days included. To further support the choice of k, Silhouette Scores were calculated separately for each value of k. When the CC procedure did not yield a conclusive value, the Silhouette Score determined the number of clusters k.

### Identification of Clinical Phenotypes

Within each identified cluster, parameters were aggregated by the mean or median as appropriate to facilitate identification and interpretation of clinical phenotypes. To determine whether clinical phenotypes differed significantly between datasets, a Kruskal-Wallis test was performed for continuous parameters, and a χ2 test for binary parameters. A Fisher's exact test was used when at least one of the expected frequencies for the binary parameters was <5.^14^ The significance level was set at 5%.

Cluster labels assigned by clustering algorithms may vary per clustering batch, making it difficult to follow clusters over time. For example, a specific cluster could be assigned label 0 at day 1 and label 2 at day 2, since the assignment of cluster labels to the actual clusters is arbitrary in k-medoids. Therefore, the assigned cluster labels were re-ordered based on their similarity over time. For this, clusters adopted the cluster label from the preceding cluster with the lowest dissimilarity value calculated as the sum of the absolute differences between the aggregated parameters of two clusters. After re-ordering the cluster labels, a Sankey diagram was created that shows the evolution of clinical phenotypes and patient allocation to these clinical phenotypes over time.

Furthermore, to identify the evolution of a single patient in a cluster over time, individual patient cluster tracks were stored as lists of cluster assignments throughout their ICU stay, up to day 21 after admission. These tracks can be either homogeneous if consisting of the same cluster only, or heterogeneous if consisting of different clusters. Comparing homogeneous with heterogeneous clusters may indicate how stable a patient is within a cluster. Note that the length of each patient's cluster track is equal to the number of days the patient is admitted (with a maximum length of 21).

## Results

**Figure 4.**
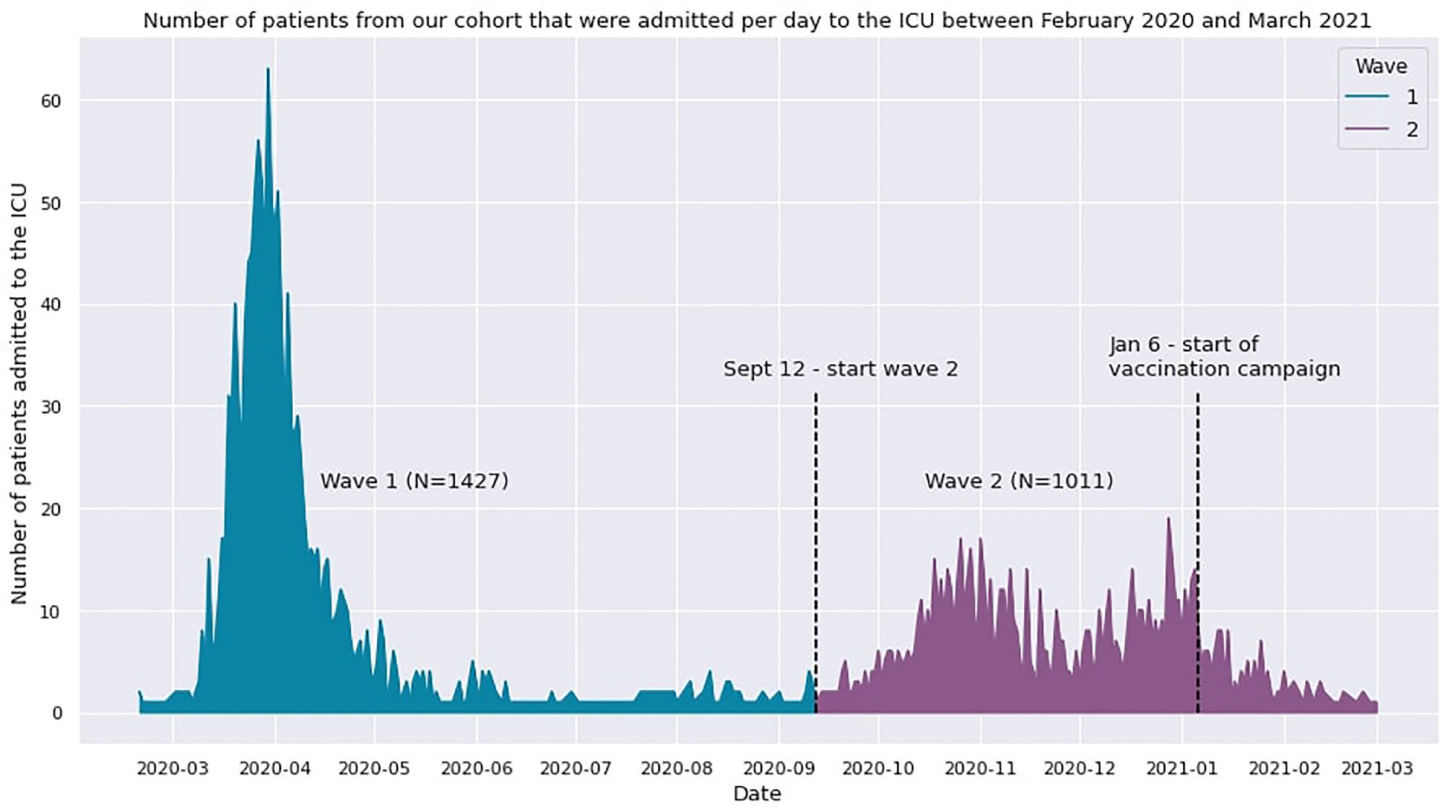
Graph that shows the admission rate per day between February 2020 and March 2021 for our patient cohort (N = 2438). During the first COVID-19 wave (until September 12, 2020), 1427 patients were admitted. From the end of the first wave to the end of our analysis (February 2021), 1011 patients were admitted to the ICU. Note that we did not include any information on COVID-19 subtypes in this figure since subtypes were not determined in the clinical setting and were thus not available in the dataset.

The final dataset included 2438 out of 3203 critically ill COVID-19 patients since these patients had a registered ICU outcome. The remaining 765 omitted patients were still admitted to the ICU at the time of data processing and were therefore not included in the analysis. The 2438 patients in the final dataset were admitted to the ICU during the first and second COVID-19 wave in the Netherlands. Figure 4 shows the number of patients that were admitted to the ICU per day during both waves, for those patients that were included in our analysis. For each patient in our analysis, an average of around 66.000 data points was available and mapped to 1331 different parameters. Of these, a selection of 135 parameters was considered clinically relevant by a team of intensivists. Subsequently, the parameters with more than 50% missing data were omitted. This resulted in a parameter set consisting of 41 parameters with, on average, 10.43% missing values per parameter (see Table A2). The parameters were categorized as either demographics, disease severity scores, cardiovascular, nephrology, respiratory, inflammatory, coagulation, or medication data (see Table A1). Cluster progression was analyzed until day 21 after ICU admission, leaving 569 patients for cluster analysis for the last day. Thus, 21 datasets with dimensions of 41 times the number of patients remaining in the ICU were available for analysis.

### Baseline Characteristics

The baseline characteristics of included patients, stratified by ICU survival status, are given in Table B1 of appendix B. Survivors were significantly younger, had lower disease severity scores, fewer indicators of organ failure, lower levels of inflammation and coagulation markers. Of note, BMI was not significantly different between survivors and non-survivors.

### Clinical Phenotypes on Admission

The first dataset was used to identify clinical phenotypes on admission. This data set included the aggregated data from the first 24 h of ICU treatment of 2438 patients. Consensus Clustering identified the ideal number of clinical phenotypes as 2. These clinical phenotypes are shown in Table 1.

**Table 1.** Characteristics of the Two Identified Clinical Phenotypes After 24 h. Note That COVID-19 Wave 1 Is Defined as the Period Before September 12, 2020.

	0 (N** **=** **931)	1 (N** **=** **1507)	Overall (N** **=** **2438)	P-Value
% admitted to the ICU in wave 1	42.4	68.5	58.5	<0.001
Outcome				
Mortality	202 (21.5%)	527 (34.6%)	729 (29.6%)	<0.001
Remaining length of ICU stay in days	10.4 (2.1, 12.9)	16.2 (6.2, 22.3)	14.0 (3.9, 18.9)	<0.001
Remaining length of hospital stay in days	17.1 (6.7, 22.8)	22.9 (10.6, 29.7)	20.9 (9.6, 28.2)	<0.001
Demographic				
Sex (female)	245 (26.1%)	404 (26.5%)	649 (26.4%)	0.853
BMI	28.8 (25.1, 31.3)	29.1 (25.4, 31.9)	29.0 (25.2, 31.6)	0.205
Age (Q1, Q3)	62.6 (56.0, 72.0)	63.7 (58.0, 72.0)	63.3 (57.0, 72.0)	0.052
Disease severity scores				
SOFA	3.2 (2.0, 4.0)	7.4 (7.0, 8.0)	5.8 (3.0, 8.0)	<0.001
APACHE	17.5 (15.0, 20.0)	20.4 (17.0, 23.0)	19.3 (16.0, 22.0)	<0.001
GCS score	14.0 (14.9, 15.0)	10.5 (4.5, 15.0)	11.8 (9.0, 15.0)	<0.001
Cardiovascular				
ABP mean (mm Hg)	85.8 (78.2, 92.7)	79.2 (74.1, 83.2)	81.7 (75.2, 86.7)	<0.001
Heart rate (/min)	82.7 (71.8, 92.7)	82.9 (71.6, 93.4)	82.8 (71.6, 93.0)	0.903
Hemoglobin (mmol/l)	7.7 (7.0, 8.5)	7.5 (6.8, 8.3)	7.6 (6.9, 8.4)	<0.001
Nephrology				
AKI	1.4 (1.0, 2.0)	1.4 (1.0, 1.5)	1.4 (1.0, 2.0)	0.093
Urea/creatinine ratio	111.05 (80.3, 130.1)	99.74 (70.9, 120.5)	104.05 (70.3, 125.8)	<0.001
Respiratory				
Invasive mechanical ventilation (%)	17.6	92.01	63.59	<0.001
PO_2_ arterial (mm Hg)	77.4 (65.6, 84.9)	82.9 (71.6, 90.0)	80.8 (69.2, 88.3)	<0.001
Mean airway pressure (cmH_2_O)	12.1 (8.7, 14.7)	16.5 (14.1, 19.0)	14.8 (11.5, 18.1)	<0.001
Peak pressure (cmH_2_O)	20.4 (14.9, 25.2)	25.8 (22.6, 29.2)	23.8 (19.1, 28.3)	<0.001
PaO_2_/FiO_2_ ratio	152.9 (97.2, 181.4)	165.6 (124.5, 193.4)	160.7 (111.8, 190.1)	<0.001
Tidal volume (ml/kg)	7.4 (6.3, 8.4)	6.9 (6.2, 7.4)	7.1 (6.2, 7.7)	<0.001
PCO_2_ arterial (mm Hg)	35.5 (31.4, 37.8)	44.9 (38.9, 49.5)	41.3 (34.5, 46.2)	<0.001
End tidal CO_2_ (mm Hg)	33.5 (29.5, 37.9)	36.6 (31.6, 41.1)	35.4 (30.8, 40.1)	<0.001
pH arterial	7.4 (7.4, 7.5)	7.4 (7.3, 7.4)	7.4 (7.4, 7.5)	<0.001
Respiratory system compliance (ml/cmH_2_O)	43.0 (27.4, 50.7)	35.4 (26.1, 41.3)	38.3 (26.5, 44.3)	<0.001
Driving pressure (cmH_2_O)	10.3 (6.0, 13.2)	12.3 (9.7, 14.5)	11.5 (8.0, 14.1)	<0.001
Respiratory rate (/min)	22.6 (18.7, 25.7)	22.5 (19.7, 25.2)	22.5 (19.4, 25.2)	0.050
Ventilatory ratio	2.0 (1.5, 2.1)	1.8 (1.4, 2.0)	1.8 (1.4, 2.1)	<0.001
Mechanical power (joules/minute/kg PBW)	0.4 (0.3, 0.5)	0.5 (0.3, 0.6)	0.4 (0.3, 0.5)	<0.001
Inflammation				
CRP (mg/l)	121.7 (53.5, 169.8)	173.5 (95.0, 243.9)	153.7 (74.0, 218.1)	<0.001
Temperature (°C)	37.1 (36.6, 37.6)	37.2 (36.6, 37.9)	37.2 (36.6, 37.8)	<0.001
Lymphocytes (%)	12.5 (6.5, 16.7)	11.0 (5.7, 14.4)	11.6 (6.0, 15.5)	<0.001
Coagulation				
APTT	33.5 (26.5, 37.0)	33.8 (27.0, 36.5)	33.7 (27.0, 36.5)	0.829
Prothrombin time (sec)	13.7 (11.1, 14.6)	13.4 (11.1, 14.3)	13.5 (11.1, 14.4)	0.178
Medication				
Anticoagulants	776 (82.7%)	1441 (94.7%)	2217 (90.1%)	<0.001
Fluids	793 (84.5%)	1461 (96.0%)	2254 (91.6%)	<0.001
Vasopressors	67 (7.1%)	1393 (91.5%)	1460 (59.3%)	<0.001
Diuretics	150 (16.0%)	310 (20.4%)	460 (18.7%)	0.008
Corticosteroids	536 (57.1%)	504 (33.1%)	1040 (42.3%)	<0.001
Other immunosuppressives	57 (6.1%)	48 (3.2%)	105 (4.3%)	0.001
Antibiotics	682 (72.7%)	1347 (88.5%)	2029 (82.5%)	<0.001
Antimycotics	66 (7.0%)	174 (11.4%)	240 (9.8%)	<0.001
Antivirals	78 (8.3%)	156 (10.2%)	234 (9.5%)	0.129
Muscle relaxants	8 (0.9%)	857 (56.3%)	865 (35.2%)	<0.001
Opioids	125 (13.3%)	1489 (97.8%)	1614 (65.6%)	<0.001
Sedatives	107 (11.4%)	1506 (98.9%)	1613 (65.6%)	<0.001

Characteristics of clinical phenotype 0 relative to clinical phenotype 1a include lower disease severity scores, lower vasopressor use, higher respiratory system compliance, lower dead space indices, lower C-reactive protein levels and administration of anticoagulants, fluids and particularly muscle relaxants, opioids and sedatives but similar P/F ratios and a higher rate of administration of corticosteroids. Of note, the number of times tocilizumab was administered was very low in both clinical phenotypes and would not have been able to affect early CRP values, implying that the difference in CRP between clinical phenotypes is real. Mortality in clinical phenotype 0 was 21.5% versus 34.6% in clinical phenotype 1a.

### Evolution of Clinical Phenotypes Over Time

**Figure 2.**
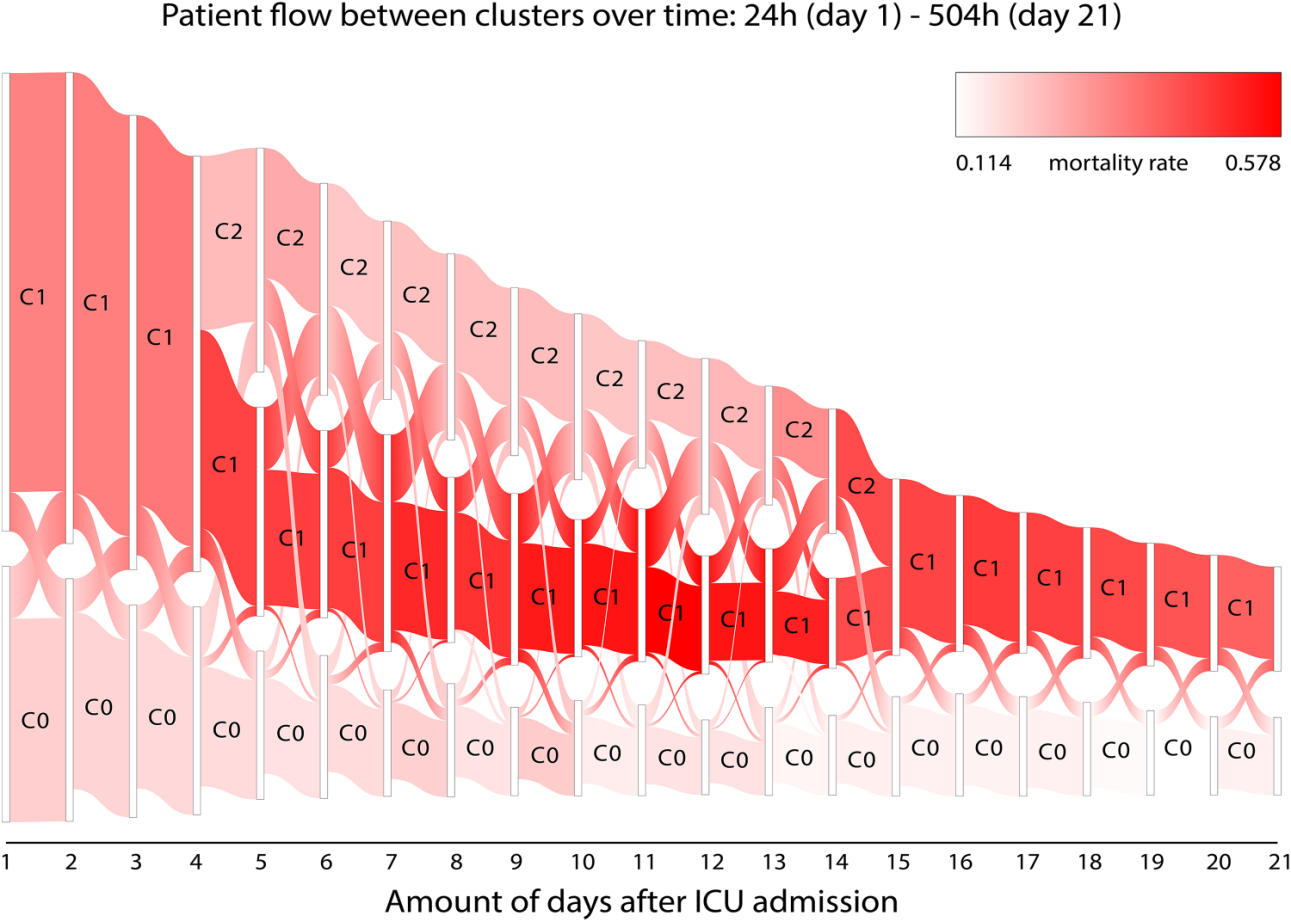
Sankey plot of the different clusters versus the amount of days (between 1 and 21) after ICU admission. Colors indicate the mortality rate of the patients in the cluster, where white represents the lowest mortality rate (0.114) and bright red depicts the highest mortality rate (0.578). The width of the cluster indicates the number of patients within the cluster. Patients switch between clusters, with an additional cluster (C2) stemming from cluster C1 from day 4 to day 13. Note that C0, C1, and C2 correspond to clinical phenotype 0, clinical phenotype 1a, and clinical phenotype 1b, respectively.

**Figure 3.**
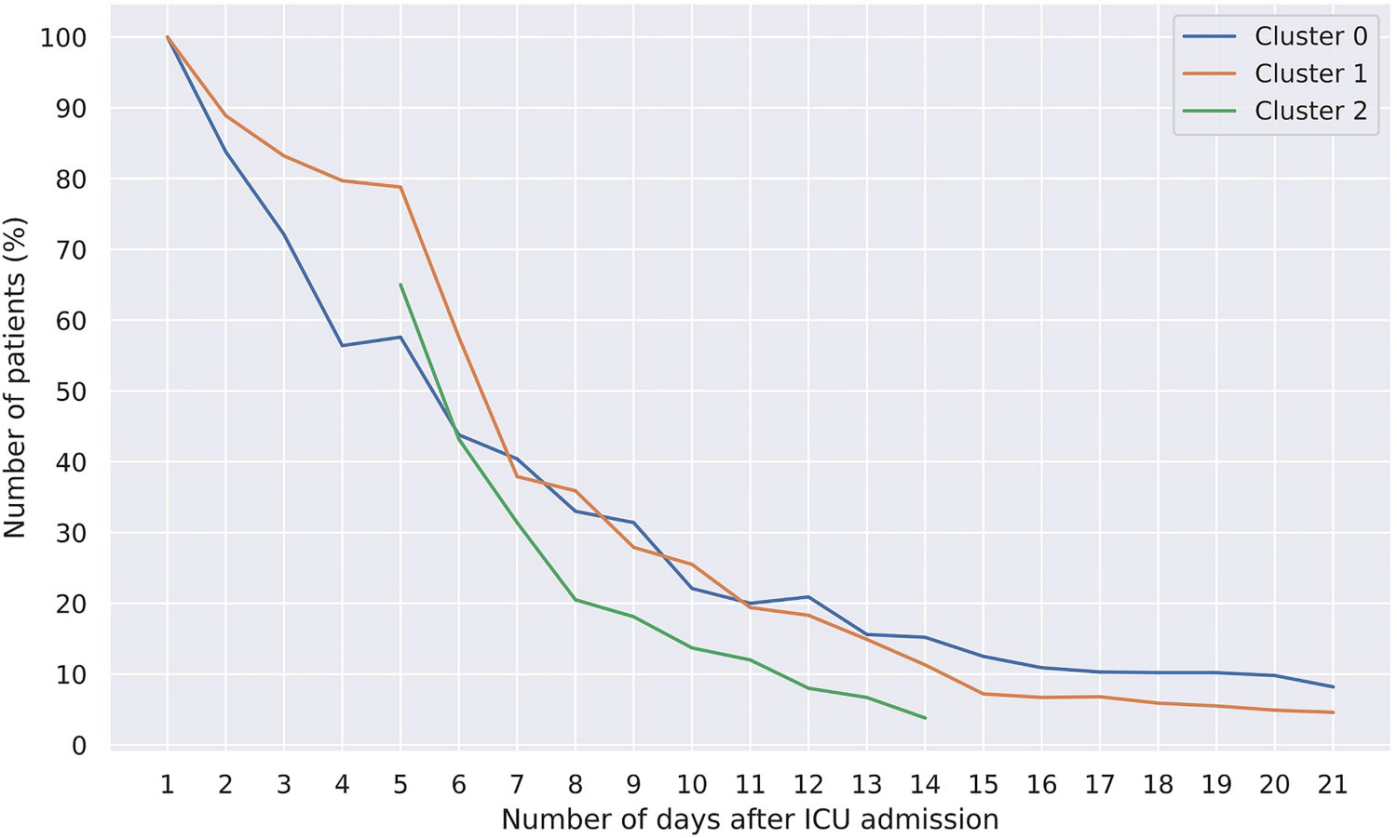
Percentage of patients remaining in their original cluster (homogeneous) over time. For example, after seven days, around 40% of the patients that started in cluster 0 have a homogeneous track up to day seven, ie they had never been in another cluster during these seven days. Please note that deceased or discharged patients are removed from the calculation, sometimes leading to an increase in the percentage within a cluster.

Figure 2 shows the evolution of clinical phenotypes and the number of allocated patients to each cluster from day 1 to day 21 of ICU treatment. Note that clusters 0, 1, and 2, correspond to phenotypes 0, 1a, and 1b, respectively. The maximum number of clusters per day was 3. An overview of characteristic of the identified clinical phenotypes at day 5 is shown in Table 2, whereas the characteristics at other time points can be found in appendix B. In addition to Figure 2, Table 3 shows the number of patients that were lost in our analysis due to discharge and death, for each day and for every cluster. Table 3 shows that the split after day 4, separating clinical phenotype 1 in clinical phenotype 1a and clinical phenotype 1b, separates a group of medium severity. In combination with Figure 2 and 3, this indicates that outcome in the worst severity group is less certain.

**Table 2.** Characteristics of the Three Identified Clinical Phenotypes at Day 5. Note That COVID-19 Wave 1 Is Defined as the Period Before September 12, 2020.

	0 (N** **=** **471)	1 (N** **=** **667)	2 (N** **=** **702)	Overall (N** **=** **1840)	P-Value
% admitted to the ICU in wave 1	46.5	76.2	58.3	61.7	<0.001
Outcome					
Mortality	86 (18.3%)	305 (45.7%)	168 (23.9%)	559 (30.4%)	<0.001
Remaining length of ICU stay in days	9.7 (1.7, 12.3)	17.1 (6.4, 23.5)	14.1 (4.3, 18.9)	14.1 (3.7, 19.7)	<0.001
Remaining length of hospital stay in days	16.41 (6.7, 22.1)	23.16 (8.9, 33.3)	21.3 (9.5, 27.9)	21.0 (8.6, 28.0)	<0.001
Demographic					
Sex (female)	119 (25.3%)	154 (23.1%)	187 (26.6%)	460 (25.0%)	0.313
BMI	29.0 (25.2, 31.5)	29.4 (25.8, 32.2)	28.6 (24.9, 31.0)	29.0 (25.2, 31.5)	0.021
Age (Q1, Q3)	63.4 (58.0, 71.0)	64.6 (59.0, 72.0)	62.7 (56.2, 71.0)	63.6 (58.0, 72.0)	0.007
Disease severity scores					
SOFA	3.5 (2.0, 4.0)	8.2 (7.0, 10.0)	5.4 (4.0, 7.0)	5.9 (3.0, 8.0)	<0.001
APACHE	17.1 (14.0, 20.0)	19.3 (16.0, 22.0)	18.1 (15.0, 21.0)	18.3 (15.0, 21.0)	<0.001
GCS score	12.8 (13.0, 15.0)	7.4 (3.0, 14.0)	10.9 (6.9, 15.0)	10.1 (3.0, 15.0)	<0.001
Cardiovascular					
ABP mean (mm Hg)	88.4 (79.7, 96.1)	78.0 (73.0, 82.6)	84.6 (77.5, 90.9)	83.2 (75.5, 89.5)	<0.001
Heart rate (/min)	84.1 (73.4, 94.5)	86.8 (74.8, 98.2)	84.1 (72.2, 95.9)	85.1 (73.4, 96.7)	0.003
Hemoglobin (mmol/l)	7.4 (6.5, 8.2)	6.7 (6.0, 7.4)	6.9 (6.2, 7.5)	7.0 (6.2, 7.7)	<0.001
Nephrology					
AKI	1.3 (1.0, 1.0)	1.6 (1.0, 2.0)	1.3 (1.0, 1.0)	1.4 (1.0, 2.0)	<0.001
Urea/creatinine ratio	149.24 (110.4, 180.1)	128.21 (80.5, 160.3)	151.3 (110.2, 190.0)	142.4 (100.9, 180.8)	<0.001
Respiratory					
Invasive mechanical ventilation (%)	40.86	96.34	86.47	78.37	<0.001
PO_2_ arterial (mm Hg)	73.9 (64.7, 80.4)	75.8 (67.4, 81.6)	76.2 (67.7, 81.8)	75.5 (66.9, 81.5)	0.001
Mean airway pressure (cmH_2_O)	12.0 (9.1, 14.3)	18.3 (15.9, 20.8)	13.9 (11.1, 16.4)	15.0 (11.5, 18.3)	<0.001
Peak pressure (cmH_2_O)	19.4 (14.2, 23.9)	28.8 (25.4, 32.0)	21.7 (17.6, 25.5)	23.7 (18.1, 28.7)	<0.001
PaO_2_/FiO_2_ ratio	166.8 (116.3, 204.2)	151.4 (117.8, 177.8)	174.0 (137.6, 201.8)	164.0 (125.3, 191.7)	<0.001
Tidal volume (ml/kg)	7.9 (6.6, 8.8)	6.8 (6.1, 7.2)	7.5 (6.4, 8.3)	7.3 (6.3, 8.1)	<0.001
PCO_2_ arterial (mm Hg)	39.6 (34.4, 42.5)	55.6 (47.5, 61.4)	45.8 (40.0, 49.6)	47.8 (39.2, 54.0)	<0.001
End tidal CO_2_ (mm Hg)	32.7 (28.9, 38.2)	40.0 (34.0, 45.6)	37.4 (32.4, 42.0)	37.2 (31.5, 42.3)	<0.001
pH arterial	7.5 (7.4, 7.5)	7.3 (7.3, 7.4)	7.4 (7.4, 7.5)	7.4 (7.4, 7.5)	<0.001
Respiratory system compliance (ml/cmH_2_O)	46.3 (28.6, 51.1)	30.5 (23.2, 35.4)	37.7 (25.0, 43.7)	37.3 (24.8, 42.4)	<0.001
Driving pressure (cmH_2_O)	8.9 (5.0, 11.9)	13.7 (10.8, 16.3)	10.1 (7.0, 12.6)	11.1 (7.5, 14.1)	<0.001
Respiratory rate (/min)	21.8 (18.0, 25.0)	25.4 (22.0, 28.4)	22.4 (18.7, 25.5)	23.3 (19.6, 26.7)	<0.001
Ventilatory ratio	1.9 (1.5, 2.1)	2.4 (1.9, 2.8)	1.9 (1.5, 2.2)	2.1 (1.6, 2.4)	<0.001
Mechanical power (joules/minute/kg PBW)	0.4 (0.2, 0.4)	0.6 (0.4, 0.7)	0.4 (0.3, 0.5)	0.5 (0.3, 0.6)	<0.001
Inflammation					
CRP (mg/l)	77.1 (13.0, 106.0)	218.1 (104.8, 321.0)	117.2 (28.0, 174.8)	143.5 (29.0, 227.0)	<0.001
Temperature (°C)	37.1 (36.6, 37.5)	37.3 (36.8, 37.9)	37.3 (36.8, 37.8)	37.3 (36.7, 37.7)	<0.001
Lymphocytes (%)	13.6 (7.4, 17.5)	11.0 (5.2, 14.7)	13.0 (6.8, 17.1)	12.4 (6.4, 16.6)	<0.001
Coagulation					
APTT	32.5 (25.0, 36.0)	35.2 (27.0, 40.0)	32.6 (25.0, 35.2)	33.5 (25.6, 37.0)	<0.001
Prothrombin time (sec)	12.8 (11.0, 14.2)	13.0 (11.0, 14.3)	12.6 (11.0, 14.0)	12.8 (11.0, 14.1)	0.114
Medication					
Anticoagulants	379 (80.5%)	643 (96.4%)	693 (98.7%)	1715 (93.2%)	<0.001
Fluids	280 (59.4%)	604 (90.6%)	586 (83.5%)	1470 (79.9%)	<0.001
Vasopressors	29 (6.2%)	576 (86.4%)	257 (36.6%)	862 (46.8%)	<0.001
Diuretics	140 (29.7%)	278 (41.7%)	327 (46.6%)	745 (40.5%)	<0.001
Corticosteroids	221 (46.9%)	169 (25.3%)	306 (43.6%)	696 (37.8%)	<0.001
Other immunosuppressives	7 (1.5%)	7 (1.0%)	8 (1.1%)	22 (1.2%)	0.788
Antibiotics	119 (25.3%)	244 (36.6%)	443 (63.1%)	806 (43.8%)	<0.001
Antimycotics	37 (7.9%)	82 (12.3%)	92 (13.1%)	211 (11.5%)	0.015
Antivirals	28 (5.9%)	60 (9.0%)	39 (5.6%)	127 (6.9%)	0.027
Muscle relaxants	5 (1.1%)	412 (61.8%)	83 (11.8%)	500 (27.2%)	<0.001
Opioids	69 (14.6%)	646 (96.9%)	666 (94.9%)	1381 (75.1%)	<0.001
Sedatives	44 (9.3%)	650 (97.5%)	657 (93.6%)	1351 (73.4%)	<0.001

**Table 3.** Number of Patients That Were Lost in Our Analysis (ie from the ICU) due to Discharge or Death, per Day and for Each Cluster. Note That This Table Starts at Day 2 (Hours 24-48) Since Between 0 and 24 h No Patients Were Lost in Our Analysis. Also Note That Cluster 0, Cluster 1, and Cluster 2 Correspond to Clinical Phenotype 1a, 1b, and 2, Respectively.

	Cluster 0		Cluster 1		Cluster 2		
Number of Days After ICU Admission	Discharge	Death	Discharge	Death	Discharge	Death	Patients Remaining
1	N/A	N/A	N/A	N/A	N/A	N/A	2438
2	114	18	47	21	N/A	N/A	2238
3	93	8	28	18	N/A	N/A	2091
4	85	6	25	20	N/A	N/A	1955
5	68	3	25	19	N/A	N/A	1840
6	88	4	8	14	24	2	1700
7	81	3	5	14	14	9	1574
8	53	2	7	21	29	4	1458
9	59	3	5	30	17	3	1341
10	51	3	1	9	22	5	1250
11	36	1	7	16	21	6	1163
12	33	3	2	16	11	3	1095
13	36	1	3	27	18	5	1005
14	36	3	2	13	17	4	930
15	38	2	4	9	5	7	865
16	29	3	4	18	N/A	N/A	811
17	34	0	5	18	N/A	N/A	754
18	26	1	6	18	N/A	N/A	703
19	23	3	7	20	N/A	N/A	650
20	18	4	1	18	N/A	N/A	609
21	20	0	1	18	0	0	570

After day 4, clinical phenotype 1b is separated from clinical phenotype 1a. Characteristics of clinical phenotype 1b relative to clinical phenotype 1a include lower disease severity scores, higher respiratory system compliance, higher P/F ratios, higher urea-to-creatinine ratio, lower C reactive protein levels and less frequent administration of vasopressors, fluids and particularly muscle relaxants, opioids and sedatives but higher rates of antibiotics and corticosteroids administration. Patients within clinical phenotype 1b on day 5 have an estimated mortality of 23.9%, which remains similar until it starts to increase a few days before it merges again with clinical phenotype 1a after day 14. In many aspects, clinical phenotype 1b can be considered the moderate version of cluster 1, which is confirmed by the high rate of crossovers between clinical phenotype 1a and clinical phenotype 1b. Even though the mortality rate of clinical phenotype 1b is comparable to that of clinical phenotype 0, very few crossovers occur between these clinical phenotypes.

Clinical phenotype 0 has the lowest mortality rate, gradually declining from 21.5% at day 1 to 13.9% at day 21. Containing approximately one third of all patients at day 1, this clinical phenotype slowly shrinks in size. A relatively large number of patients in cluster 0 cross over to cluster 1 and vice versa during the first four days. After that, cluster 0 appears to be relatively stable with only small numbers of patients crossing over to a different cluster or vice versa.

Clinical phenotype 1a has the highest mortality rate, starting at 34.6% at day 1. After separation of clinical phenotype 1b at day 4, mortality in clinical phenotype 1a rises progressively to 55% between day 10 and day 13. After day 13, mortality gradually declines again to around 40% at day 21. Clinical phenotype 1a remains the largest clinical phenotype for most of the 21-day period.

### Clinical Phenotype Homogeneity

Figure 3 gives an overview of the number of patients with a homogeneous track on each day until day 21. Patients in clinical phenotype 1a are more stable in terms of their clinical phenotype assignments between day one and day four, compared to clinical phenotype 0. Due to the separation of clinical phenotype 1a into clinical phenotype 1a and clinical phenotype 1b, all three clinical phenotypes show a similar declining trend in the number of homogeneous patients left after day 5. After 21 days, only 8.2% (20/243) and 4.6% (15/327) of the patients were still homogeneous for clinical phenotype 0 and 1a, respectively.

## Discussion

This is the first study to identify the evolution of clinical phenotypes as well as patient allocation to these clinical phenotypes over time throughout the course of intensive care treatment. Our main findings are that in our cohort of critically ill COVID-19 patients, the identified clinical phenotypes do not remain stable but rather evolve over time, and that patients typically do not remain in the same cluster but rather cross back and forward between clusters over time. This indicates an improvement or decline throughout their intensive care treatment, which is not reflected in the static disease assessment scores taken on admission. Furthermore, this indicates that patients with an initial bad severity score can still have good outcomes. It would be interesting for future research to evaluate if a prediction model can be trained that is able to sequentially assess cluster switching.

This novel finding of clinical phenotype instability may be best understood by introducing the concept of clinical phenotype half-life. This is defined as the time that an identified clinical phenotype has lost half of its allocated patients. Figure 3 reveals the clinical phenotype half-life to be between 5 and 6 days for clinical phenotypes 0 and 1a respectively, and about 3 days for clinical phenotype 1b. For our cohort, for example, it may be argued that clinical phenotype-based prognostication changes for about half of the patients within one week. Similarly, if a predictive enrichment strategy would lead to the enrollment of patients of a specific clinical phenotype identified on admission based on presumed benefit in that cluster, this presumed benefit would not last in over about half of these patients after one week as they have crossed over to a different cluster. Short to very short clinical phenotype half-life may have important implications for the use of clinical phenotyping on admission for prognostic or predictive enrichment.

In our study, up to three distinct clinical phenotypes were identified by cluster analyses, each with different mortality rates and characteristics. Only two clinical phenotypes could be identified at the time of ICU admission, with one that splits into two separate clinical phenotypes over time. At first sight, the separation in two clinical phenotypes on ICU admission appears to be largely driven by the need for invasive mechanical ventilation and hence explain the large differences in opioid administration and muscle relaxants. While it is reassuring that the algorithm identifies this heterogeneity, separation based on type of respiratory support would obviously be of limited clinical utility as it is easily observed at the bedside. Clinical phenotype 1a may be characterized as hyperinflammatory and has higher levels of dead space ventilation, possibly indicative of undiagnosed pulmonary microclots or pulmonary embolism. These findings coincide with less frequent administration of corticosteroids and anticoagulants. The further separation of clinical phenotype 1b from clinical phenotype 1a reveals similar insights. Again, the heterogeneity appears to be strongly driven by inflammation and dead space ventilation, also with coinciding findings of less frequent administration of corticosteroids and anticoagulants. Taken together, these findings may confirm the previously underestimated role of coagulation and specifically undiagnosed pulmonary microclots or pulmonary embolism as well as the beneficial effects of steroids, and suggests that suppression of inflammation may also be relevant at later stages of the disease. These evaluations of different clusters may help in future research to identify the reasons why patients switch between clusters and set up studies which seek out how to obtain the optimal outcome in patients.

It is difficult to compare our results to other clustering efforts given the different patient cohorts that were considered, ie hospitalized versus admitted to intensive care.^3, 4^ However, Rodríguez et al^5^ only included intensive care patients for clustering and also found three distinct COVID-19 clinical phenotypes: a mild one, a moderate one, and a severe one. Their mild clinical phenotype has a crude mortality of 20.3%, which seems to correspond reasonably well to our clinical phenotype 0 in terms of mortality rate and some clinical characteristics, but the moderate and severe ones do not. However, these results are again difficult to compare with our findings due to different parameter sets to describe the clinical phenotypes, for example the inclusion of comorbidities and coexisting conditions and the use of different laboratory findings and treatments.

Bos et al performed cluster analysis on a similar patient cohort to identify respiratory subphenotypes of COVID-19 ARDS.^15^ Their analysis included longitudinal clusters that incorporated data points during the first four days of admission which they found to discriminate and prognosticate better than only considering data on admission. This confirms the importance of including a temporal dimension in clinical phenotyping. However, our approach is different as we did not exclusively focus on respiratory data and determined clinical phenotypes for each single day of admission instead of grouping data over multiple days. By phenotyping beyond day four, our approach indeed revealed the emergence of a clinical phenotype beyond the first four days of admission.

Our study comes with limitations. First, this study used 41 parameters to describe the clinical phenotypes, which is relatively high and may impair the understanding of the relationship of the variables to the outcome.^16^ Second, several parameters had a relatively high proportion of missing values (>30%) that were consequently imputed. Imputing missing data is undoubtedly important, but also difficult and complex. There is no gold standard for comparison which hampers the quality evaluation of the imputations. For these reasons, our clinical phenotypes should be interpreted with caution. Third, all of the 21 data sets that were created covered a 24-h period. The length of this period decides the granularity of the clinical phenotypes and the level of interaction between those clinical phenotypes. A higher granularity in the data sets, eg 8-h periods instead of 24-h periods may provide a higher level of detail within the identified clinical phenotypes. Future research should focus on testing different granularities, to determine which resolution offer the best clinical insights. Fourth, in our analysis we included patients that were admitted to the ICU between February 2020 and March 2021. Due to the length of this period, our results may have been influenced by different COVID-19 variants and implementation of new treatment strategies. We were not able to verify this since COVID-19 subtypes were not determined in the clinical setting and were thus not available for analysis. In addition, symptom duration and onset were not recorded and thus not taken into account, while this may provide valuable insight. Furthermore, vaccines were made available on the sixth of January 2021, prioritizing high risk patients and healthcare workers. We expect vaccines to have had minimal impact on our analyses, given the low admission rates for January, February and March 2021. However, we could not validate this assumption since vaccination status was not systematically recorded and therefore not available in our datasets. Lastly, this study is limited by the lack of a validation cohort. However, this is unavoidable due to the uniqueness of the data set used for this study.

## Conclusions

Clinical phenotypes in critically ill COVID-19 patients do not remain stable and patients typically do not remain in the same cluster throughout intensive care treatment. Short to very short clinical phenotype half-life may have important implications for the use of clinical phenotyping on admission for prognostic or predictive enrichment. The heterogeneity between the identified clinical phenotypes seems to be strongly driven by markers of inflammation and the level of dead space ventilation.

## List of abbreviations

ABP: Arterial Blood Pressure
AKI: Acute Kidney Injury
APACHE: Acute Physiology and Chronic Health Evaluation
APTT: Activated Partial Thromboplastin Time
ARDS: Acute Respiratory Distress Syndrome
BMI: Body Mass Index
CC: Consensus Clustering
CDF: Cumulative distribution function
CRP: C-reactive Protein
DDW: Dutch ICU Data Warehouse
FiO_2_: Fraction of inspired oxygen
GCS: Glasgow Coma Scale
ICU: Intensive Care Unit
MICE: Multiple Imputations by Chained Equations
PAM: Partitioning around Medoids
PBW: Predicted body weight
PO_2_: Partial pressure O_2_
SOFA: Sequential Organ Failure Assessment
SS: Silhouette Scores

## Appendices

### Appendix A

**Table A1.** The Included Parameters in the Data Sets with Their Descriptions, Additional Information, and Variable Type.

Parameter	Description	Comment	Variable Type
Sex (female)	Percentage of female patients	N/A	Categorical
BMI	Body Mass Index	N/A	Numerical
Age (Q1, Q3)	Median age with 25^th^ and 75^th^ percentiles	N/A	Numerical
SOFA	Total SOFA score	Ignores missing components	Numerical
APACHE	Total APACHE II score	Ignores missing components	Numerical
GCS score	Total Glasgow Coma Scale-score	N/A	Numerical
ABP mean (mm Hg)	Arterial blood pressure (mean)	N/A	Numerical
Heart rate (/min)	Heart rate	Either ECG, unspecified, or monitor	Numerical
Hemoglobin (mmol/l)	Hemoglobin	Either arterial, blood, mixed venous, unspecified, venous, or bloodgas	Numerical
AKI	The creatinine component of the AKI (Acute Kidney Injury) score	N/A	Numerical
Urea/creatinine ratio	Urea over creatinine	N/A	Numerical
PO_2_ arterial (mm Hg)	Partial pressure O2 (arterial)	N/A	Numerical
Mean airway pressure (cmH_2_O)	Mean airway pressure	N/A	Numerical
Peak pressure (cmH_2_O)	Peak pressure	N/A	Numerical
PaO_2_/FiO_2_	PaO_2_/FiO_2_ ratio	N/A	Numerical
Tidal volume (ml/kg)	Tidal volume per ideal body weight	N/A	Numerical
PCO_2_ arterial (mm Hg)	Partial pressure CO_2_ (arterial)	N/A	Numerical
End tidal CO_2_ (mm Hg)	End-tidal CO_2_	N/A	Numerical
pH arterial	pH arterial	N/A	Numerical
Respiratory system compliance (ml/cmH_2_O)	Respiratory system compliance	Either dynamic or unspecified	Numerical
Driving pressure (cmH_2_O)	Driving pressure	N/A	Numerical
Respiratory rate (/min)	Respiratory rate	Measured by ventilator	Numerical
Ventilatory ratio	Ventilatory ratio	Calculated as: (minute ventilation (ml/min) · PaCO_2_ (mm Hg))/(predicted body weight · 100 · 37.5)	Numerical
Mechanical power (joules/minute/kg PBW)	Mechanical power per predicted body weight	N/A	Numerical
CRP (mg/l)	C-reactive protein	Either blood or unspecified	Numerical
Temperature (°C)	Temperature in degrees Celsius	Either unspecified, rectal, esophageal, nasopharyngeal, groin, bladder, core, blood, axillary, or ear	Numerical
Lymphocytes (%)	Lymphocytes percentage	N/A	Numerical
APTT	Activated partial thromboplastin time	Either blood or unspecified	Numerical
Prothrombin time (sec)	Prothrombin time	N/A	Numerical
Anticoagulants	Percentage of patients with anticoagulants intake	Includes medications: heparin, dalteparin, enoxaparin, nadroparin, danaparoid, tinzaparin, clopidogrel, dipyridamole, carbasalate, epoprostenol, ticagrelor	Categorical
Fluids	Percentage of patients with fluids intake	Includes medications: erythrocytes, thrombocytes, blood, solutions, carbohydrates, electrolytes	Categorical
Vasopressors	Percentage of patients with vasopressors intake	Includes medications: isoprenaline, norepinephrine, dopamine, phenylephrine, dobutamine, epinephrine, ephedrine, milrinone, enoximone, vasopressin, desmopressin	Categorical
Diuretics	Percentage of patients with diuretics intake	Includes medications: furosemide, bumetanide	Categorical
Corticosteroids	Percentage of patients with corticosteroids intake	Includes medications: corticosteroids hydrocortisone, prednisolone, betamethasone, dexamethasone, betamethasone, dexamethasone, methylprednisolone, prednisolone, prednisone, hydrocortisone, cortisone, methylprednisolone	Categorical
Other immunosuppressives	Percentage of patients with other immunosuppressives intake	Includes medications: anakinra, tocilizumab	Categorical
Antibiotics	Percentage of patients with antibiotics intake	Includes medications: flucloxacillin, cefuroxime, cefotaxime, ceftazidime, ceftriaxone, meropenem, gentamicin, ciprofloxacin, vancomycin, metronidazole	Categorical
Antimycotics	Percentage of patients with antimycotics intake	Includes medications: amphotericin, miconazole, ketoconazole, fluconazole, voriconazole, anidulafungin	Categorical
Antivirals	Percentage of patients with antivirals intake	Includes medicationcyclovirvir, ganciclovir, valaciclovir, valganciclovir, ritonavir, oseltamivir, lopinavir, remdesivir	Categorical
Musclerelaxants	Percentage of patients with musclerelexants intake	Includes medications: suxamethonium, rocuronium	Categorical
Opioids	Percentage of patients with opioids intake	Includes medications: fentanyl, alfentanil, sufentanil, remifentanil, morphine, fentanyl, piritramide	Categorical
Sedatives	Percentage of patients with sedatives intake	Includes medications: ketamine, etomidate, propofol, esketamine, diazepam, lorazepam, midazolam, dexmedetomidine	Categorical

**Table A2.** Percentage of Missing Values for Each Parameter That Was Included in the Analyses. Note That These Are the Percentages Missing Values in the First Dataset (Hours 0-24 After ICU Admission). The Other Datasets (Hours 24-504) Have Fewer Missing Values for Each Parameter.

Parameter	Percentage (%) Missing Values
Sex (female)	0.1787
BMI	8.5344
Age (Q1, Q3)	0.0893
SOFA	0.3128
APACHE	0.0000
GCS score	4.0661
ABP mean (mm Hg)	2.7703
Heart rate (/min)	3.2618
Hemoglobin (mmol/l)	2.8596
AKI	36.3003
Urea/creatinine ratio	5.6319
PO_2_ arterial (mm Hg)	9.2493
Mean airway pressure (cmH_2_O)	32.1715
Peak pressure (cmH_2_O)	18.6327
PaO_2_/FiO_2_	11.8409
Tidal volume (ml/kg)	17.6497
PCO_2_ arterial (mm Hg)	14.9683
End tidal CO_2_ (mm Hg)	21.2243
pH arterial	16.1784
Respiratory system compliance (ml/cmH_2_O)	22.2073
Driving pressure (cmH_2_O)	22.4307
Respiratory rate (/min)	27.3011
Ventilatory ratio	28.0608
Mechanical power (joules/minute/kg PBW)	30.3842
CRP (mg/l)	3.1277
Temperature (°C)	2.0554
Lymphocytes (%)	27.8373
APTT	17.1134
Prothrombin time (sec)	31.8588
Anticoagulants	0.0000
Fluids	0.0000
Vasopressors	0.0000
Diuretics	0.0000
Corticosteroids	0.0000
Other immunosuppressives	0.0000
Antibiotics	0.0000
Antimycotics	0.0000
Antivirals	0.0000
Musclerelaxants	0.0000
Opioids	0.0000
Sedatives	0.0000

### Appendix B

**Table B1.** Baseline Characteristics at the 24-h Mark After Admission, Stratified by Mortality Outcome. Note That COVID-19 Wave 1 Is Defined as the Period Before September 12, 2020.

	Survivor (N = 1716)	Non-survivor(N = 722)	Overall (N = 2438)	P-Value
% admitted to the ICU in wave 1	58.3	58.9	58.5	0.398
Outcome				
Remaining length of ICU stay in days	13.1 (2.8, 17.7)	12.7 (3.6, 18.2)	13.0 (2.9, 17.9)	0.077
Demographic				
Sex (female)	479 (27.7%)	170 (23.3%)	649 (26.4%)	0.029
BMI	29.0 (25.3, 31.6)	28.9 (25.2, 31.9)	29.0 (25.2, 31.6)	0.597
Age (Q1, Q3)	61.1 (55.0, 70.0)	68.6 (64.0, 75.0)	63.3 (57.0, 72.0)	<0.001
Disease severity scores				
SOFA	5.4 (3.0, 7.0)	6.9 (4.0, 9.0)	5.8 (3.0, 8.0)	<0.001
APACHE	18.6 (16.0, 22.0)	20.9 (18.0, 24.0)	19.3 (16.0, 22.0)	<0.001
GCS score	12.5 (11.0, 15.0)	10.1 (3.0, 15.0)	11.8 (9.0, 15.0)	<0.001
Cardiovascular				
ABP mean (mm Hg)	82.7 (75.9, 88.0)	79.3 (73.5, 84.4)	81.7 (75.2, 86.7)	<0.001
Heart rate (/min)	82.1 (71.0, 92.2)	84.6 (72.6, 95.3)	82.8 (71.6, 93.0)	<0.001
Hemoglobin (mmol/l)	7.7 (7.0, 8.4)	7.5 (6.7, 8.3)	7.6 (6.9, 8.4)	0.002
Nephrology				
AKI	1.3 (1.0, 1.0)	1.6 (1.0, 2.0)	1.4 (1.0, 2.0)	<0.001
Urea/creatinine ratio	102.94 (70.2, 124.6)	107.0 (75.8, 130.8)	104.13 (70.7, 127.0)	0.015
Respiratory				
Invasive mechanical ventilation (%)	60.29	71.55	63.62	<0.001
PO_2_ arterial (mm Hg)	81.0 (69.0, 88.8)	80.4 (69.3, 87.0)	80.8 (69.2, 88.3)	0.516
Mean airway pressure (cmH_2_O)	14.2 (11.0, 17.5)	16.2 (13.4, 19.0)	14.8 (11.5, 18.1)	<0.001
Peak pressure (cmH_2_O)	22.8 (18.0, 27.4)	26.1 (22.6, 30.4)	23.8 (19.1, 28.3)	<0.001
PaO_2_/FiO_2_ ratio	163.5 (113.1, 194.1)	154.2 (109.6, 182.7)	160.7 (111.8, 190.1)	0.002
Tidal volume (ml/kg)	7.2 (6.3, 7.7)	6.9 (6.1, 7.7)	7.1 (6.2, 7.7)	0.007
PCO_2_ arterial (mm Hg)	40.3 (34.2, 45.0)	43.6 (35.8, 49.5)	41.3 (34.5, 46.2)	<0.001
End tidal CO_2_ (mm Hg)	35.1 (30.8, 39.8)	36.2 (30.4, 41.0)	35.4 (30.8, 40.1)	0.576
pH arterial	7.4 (7.4, 7.5)	7.4 (7.3, 7.4)	7.4 (7.4, 7.5)	<0.001
Respiratory system compliance (ml/cmH_2_O)	40.3 (28.4, 46.4)	33.4 (23.3, 39.7)	38.3 (26.5, 44.3)	<0.001
Driving pressure (cmH_2_O)	10.8 (8.0, 13.5)	13.1 (9.8, 16.0)	11.5 (8.0, 14.1)	<0.001
Respiratory rate (/min)	22.3 (19.3, 25.0)	23.2 (19.7, 26.1)	22.5 (19.4, 25.2)	<0.001
Ventilatory ratio	1.8 (1.4, 2.0)	2.0 (1.5, 2.3)	1.8 (1.4, 2.1)	<0.001
Mechanical power (joules/minute/kg PBW)	0.4 (0.3, 0.5)	0.5 (0.3, 0.6)	0.4 (0.3, 0.5)	<0.001
Inflammation				
CRP (mg/l)	145.1 (66.8, 208.0)	174.3 (92.5, 239.5)	153.7 (74.0, 218.1)	<0.001
Temperature (°C)	37.2 (36.6, 37.8)	37.2 (36.6, 37.8)	37.2 (36.6, 37.8)	0.947
Lymphocytes (%)	12.6 (6.5, 16.7)	9.2 (4.7, 11.8)	11.6 (6.0, 15.5)	<0.001
Coagulation				
APTT	32.7 (26.5, 35.2)	36.1 (27.5, 40.5)	33.7 (27.0, 36.5)	<0.001
Prothrombin time (sec)	13.1 (11.0, 14.0)	14.4 (11.4, 15.0)	13.5 (11.1, 14.4)	<0.001
Medication				
Anticoagulants	1547 (89.4%)	670 (91.9%)	2217 (90.1%)	0.064
Fluids	1569 (90.6%)	685 (94.0%)	2254 (91.6%)	0.008
Vasopressors	942 (54.4%)	518 (71.1%)	1460 (59.3%)	<0.001
Diuretics	307 (17.7%)	153 (21.0%)	460 (18.7%)	0.067
Corticosteroids	736 (42.5%)	304 (41.7%)	1040 (42.3%)	0.741
Other immunosuppressives	87 (5.0%)	18 (2.5%)	105 (4.3%)	0.006
Antibiotics	1409 (81.4%)	620 (85.0%)	2029 (82.5%)	0.034
Antimycotics	162 (9.4%)	78 (10.7%)	240 (9.8%)	0.343
Antivirals	158 (9.1%)	76 (10.4%)	234 (9.5%)	0354
Muscle relaxants	572 (33.0%)	293 (40.2%)	865 (35.2%)	0.001
Opioids	1073 (62.0%)	541 (74.2%)	1614 (65.6%)	<0.001
Sedatives	1066 (61.6%)	547 (75.0%)	1613 (65.6%)	<0.001

**Table B2.** Characteristics of the Two Identified Clinical Phenotypes at Day 1. Note That COVID-19 Wave 1 Is Defined as the Period Before September 12, 2020.

	0 (N = 931)	1 (N** **=** **1507)	Overall (N** **=** **2438)	P-Value
% admitted to the ICU in wave 1	42.4	68.5	58.5	<0.001
Outcome				
Mortality	202 (21.5%)	527 (34.6%)	729 (29.6%)	<0.001
Remaining length of ICU stay in days	10.4 (2.1, 12.9)	16.2 (6.2, 22.3)	14.0 (3.9, 18.9)	<0.001
Remaining length of hospital stay in days	17.1 (6.7, 22.8)	22.9 (10.6, 29.7)	20.9 (9.6, 28.2)	<0.001
Demographic				
Sex (female)	245 (26.1%)	404 (26.5%)	649 (26.4%)	0.853
BMI	28.8 (25.1, 31.3)	29.1 (25.4, 31.9)	29.0 (25.2, 31.6)	0.205
Age (Q1, Q3)	62.6 (56.0, 72.0)	63.7 (58.0, 72.0)	63.3 (57.0, 72.0)	0.052
Disease severity scores				
SOFA	3.2 (2.0, 4.0)	7.4 (7.0, 8.0)	5.8 (3.0, 8.0)	<0.001
APACHE	17.5 (15.0, 20.0)	20.4 (17.0, 23.0)	19.3 (16.0, 22.0)	<0.001
GCS score	14.0 (14.9, 15.0)	10.5 (4.5, 15.0)	11.8 (9.0, 15.0)	<0.001
Cardiovascular				
ABP mean (mm Hg)	85.8 (78.2, 92.7)	79.2 (74.1, 83.2)	81.7 (75.2, 86.7)	<0.001
Heart rate (/min)	82.7 (71.8, 92.7)	82.9 (71.6, 93.4)	82.8 (71.6, 93.0)	0.903
Hemoglobin (mmol/l)	7.7 (7.0, 8.5)	7.5 (6.8, 8.3)	7.6 (6.9, 8.4)	<0.001
Nephrology				
AKI	1.4 (1.0, 2.0)	1.4 (1.0, 1.5)	1.4 (1.0, 2.0)	0.093
Urea/creatinine ratio	111.05 (80.3, 130.1)	99.74 (70.9, 120.5)	104.05 (70.3, 125.8)	<0.001
Respiratory				
Invasive mechanical ventilation (%)	17.6	92.01	63.59	<0.001
PO_2_ arterial (mm Hg)	77.4 (65.6, 84.9)	82.9 (71.6, 90.0)	80.8 (69.2, 88.3)	<0.001
Mean airway pressure (cmH_2_O)	12.1 (8.7, 14.7)	16.5 (14.1, 19.0)	14.8 (11.5, 18.1)	<0.001
Peak pressure (cmH_2_O)	20.4 (14.9, 25.2)	25.8 (22.6, 29.2)	23.8 (19.1, 28.3)	<0.001
PaO_2_/FiO_2_ ratio	152.9 (97.2, 181.4)	165.6 (124.5, 193.4)	160.7 (111.8, 190.1)	<0.001
Tidal volume (ml/kg)	7.4 (6.3, 8.4)	6.9 (6.2, 7.4)	7.1 (6.2, 7.7)	<0.001
PCO_2_ arterial (mm Hg)	35.5 (31.4, 37.8)	44.9 (38.9, 49.5)	41.3 (34.5, 46.2)	<0.001
End tidal CO_2_ (mm Hg)	33.5 (29.5, 37.9)	36.6 (31.6, 41.1)	35.4 (30.8, 40.1)	<0.001
pH arterial	7.4 (7.4, 7.5)	7.4 (7.3, 7.4)	7.4 (7.4, 7.5)	<0.001
Respiratory system compliance (ml/cmH_2_O)	43.0 (27.4, 50.7)	35.4 (26.1, 41.3)	38.3 (26.5, 44.3)	<0.001
Driving pressure (cmH_2_O)	10.3 (6.0, 13.2)	12.3 (9.7, 14.5)	11.5 (8.0, 14.1)	<0.001
Respiratory rate (/min)	22.6 (18.7, 25.7)	22.5 (19.7, 25.2)	22.5 (19.4, 25.2)	0.050
Ventilatory ratio	2.0 (1.5, 2.1)	1.8 (1.4, 2.0)	1.8 (1.4, 2.1)	<0.001
Mechanical power (joules/minute/kg PBW)	0.4 (0.3, 0.5)	0.5 (0.3, 0.6)	0.4 (0.3, 0.5)	<0.001
Inflammation				
CRP (mg/l)	121.7 (53.5, 169.8)	173.5 (95.0, 243.9)	153.7 (74.0, 218.1)	<0.001
Temperature (°C)	37.1 (36.6, 37.6)	37.2 (36.6, 37.9)	37.2 (36.6, 37.8)	<0.001
Lymphocytes (%)	12.5 (6.5, 16.7)	11.0 (5.7, 14.4)	11.6 (6.0, 15.5)	<0.001
Coagulation				
APTT	33.5 (26.5, 37.0)	33.8 (27.0, 36.5)	33.7 (27.0, 36.5)	0.829
Prothrombin time (sec)	13.7 (11.1, 14.6)	13.4 (11.1, 14.3)	13.5 (11.1, 14.4)	0.178
Medication				
Anticoagulants	776 (82.7%)	1441 (94.7%)	2217 (90.1%)	<0.001
Fluids	793 (84.5%)	1461 (96.0%)	2254 (91.6%)	<0.001
Vasopressors	67 (7.1%)	1393 (91.5%)	1460 (59.3%)	<0.001
Diuretics	150 (16.0%)	310 (20.4%)	460 (18.7%)	0.008
Corticosteroids	536 (57.1%)	504 (33.1%)	1040 (42.3%)	<0.001
Other immunosuppressives	57 (6.1%)	48 (3.2%)	105 (4.3%)	0.001
Antibiotics	682 (72.7%)	1347 (88.5%)	2029 (82.5%)	<0.001
Antimycotics	66 (7.0%)	174 (11.4%)	240 (9.8%)	<0.001
Antivirals	78 (8.3%)	156 (10.2%)	234 (9.5%)	0.129
Muscle relaxants	8 (0.9%)	857 (56.3%)	865 (35.2%)	<0.001
Opioids	125 (13.3%)	1489 (97.8%)	1614 (65.6%)	<0.001
Sedatives	107 (11.4%)	1506 (98.9%)	1613 (65.6%)	<0.001

**Table B3.** Characteristics of the Two Identified Clinical Phenotypes at Day 14. Note That COVID-19 Wave 1 Is Defined as the Period Before September 12, 2020.

	0 (N = 250)	1 (N** **=** **286)	2 (N** **=** **394)	Overall (N** **=** **930)	P-Value
% admitted to the ICU in wave 1	69.6	64.4	71.4	68.8	<0.001
Outcome					
Mortality	33 (13.2%)	149 (52.1%)	125 (31.7%)	307 (33.0%)	<0.001
Remaining length of ICU stay in days	11.7 (1.8, 15.0)	16.3 (4.4, 22.9)	15.9 (5.7, 21.2)	14.9 (4.1, 20.9)	<0.001
Remaining length of hospital stay in days	19.7 (9.9, 25.1)	21.8 (4.4, 37.6)	25.5 (11.5, 35.8)	22.9 (8.8, 33.1)	<0.001
Demographic					
Sex (female)	71 (28.4%)	57 (19.9%)	99 (25.1%)	227 (24.4%)	0.068
BMI	28.5 (24.5, 30.7)	28.0 (24.5, 30.5)	29.1 (25.5, 31.6)	28.6 (24.9, 31.0)	0.055
Age (Q1, Q3)	63.4 (58.0, 71.0)	64.7 (59.0, 72.0)	63.7 (58.0, 72.0)	63.9 (58.0, 72.0)	0.558
Disease severity scores					
SOFA	3.4 (2.0, 4.0)	8.1 (7.0, 10.0)	5.7 (4.0, 7.0)	5.8 (3.0, 8.0)	<0.001
APACHE	17.8 (15.0, 20.0)	21.0 (18.0, 24.0)	20.1 (17.0, 23.0)	19.7 (17.0, 23.0)	<0.001
GCS score	13.0 (13.0, 15.0)	7.0 (3.0, 14.0)	10.0 (4.0, 15.0)	9.9 (3.0, 15.0)	<0.001
Cardiovascular					
ABP mean (mm Hg)	86.4 (78.1, 94.2)	77.3 (71.9, 81.7)	82.3 (74.6, 88.9)	81.9 (74.1, 88.1)	<0.001
Heart rate (/min)	92.0 (81.1, 101.4)	91.7 (82.2, 101.8)	91.5 (80.1, 102.1)	91.7 (80.9, 101.8)	0.821
Hemoglobin (mmol/l)	6.5 (5.7, 7.1)	5.9 (5.2, 6.4)	6.0 (5.3, 6.6)	6.1 (5.3, 6.7)	<0.001
Nephrology					
AKI	1.3 (1.0, 1.0)	1.6 (1.0, 2.9)	1.4 (1.0, 2.0)	1.4 (1.0, 2.0)	<0.001
Urea/creatinine ratio	182.9 (140.1, 225.0)	167.13 (100.2, 210.9)	180.0 (130.7, 230.3)	176.86 (120.0, 220.4)	0.003
Respiratory					
Invasive mechanical ventilation (%)	44.8	81.63	78.01	70.19	<0.001
PO_2_ arterial (mm Hg)	79.0 (67.0, 85.9)	75.3 (67.1, 80.1)	77.0 (67.2, 83.0)	77.0 (67.1, 83.0)	0.051
Mean airway pressure (cmH_2_O)	11.6 (9.0, 13.3)	16.7 (14.2, 19.5)	13.7 (11.0, 15.7)	14.1 (10.6, 17.1)	<0.001
Peak pressure (cmH_2_O)	18.2 (13.0, 21.3)	28.5 (24.4, 32.5)	22.1 (17.4, 26.7)	23.0 (16.8, 28.9)	<0.001
PaO_2_/FiO_2_ ratio	217.6 (154.8, 260.0)	153.5 (109.4, 177.8)	178.8 (135.3, 212.6)	181.5 (129.8, 216.9)	<0.001
Tidal volume (ml/kg)	7.5 (6.5, 8.8)	6.6 (5.9, 7.3)	7.8 (6.7, 8.7)	7.3 (6.3, 8.2)	<0.001
PCO_2_ arterial (mm Hg)	39.5 (34.0, 42.6)	58.2 (47.0, 66.7)	47.8 (39.9, 52.5)	48.8 (38.7, 55.9)	<0.001
End tidal CO_2_ (mm Hg)	33.3 (29.0, 38.3)	41.2 (33.8, 47.5)	37.5 (30.7, 42.8)	37.5 (30.9, 43.0)	<0.001
pH arterial	7.5 (7.4, 7.5)	7.4 (7.3, 7.4)	7.4 (7.4, 7.5)	7.4 (7.4, 7.5)	<0.001
Respiratory system compliance (ml/cmH_2_O)	40.7 (26.1, 46.4)	26.3 (17.4, 30.5)	34.6 (22.2, 41.0)	33.7 (21.0, 40.1)	<0.001
Driving pressure (cmH_2_O)	8.8 (5.1, 12.0)	15.0 (11.4, 18.5)	11.2 (7.6, 14.2)	11.7 (7.4, 15.7)	<0.001
Respiratory rate (/min)	25.2 (20.5, 29.3)	26.1 (22.5, 29.7)	25.2 (22.0, 28.6)	25.5 (21.9, 29.1)	0.029
Ventilatory ratio	1.9 (1.6, 2.3)	2.4 (1.9, 2.9)	2.3 (1.9, 2.6)	2.2 (1.8, 2.6)	<0.001
Mechanical power (joules/minute/kg PBW)	0.4 (0.3, 0.5)	0.5 (0.4, 0.6)	0.5 (0.4, 0.6)	0.5 (0.3, 0.6)	<0.001
Inflammation					
CRP (mg/l)	71.2 (17.2, 94.8)	159.6 (68.2, 240.0)	112.2 (40.0, 168.5)	115.8 (34.0, 167.0)	<0.001
Temperature (°C)	37.3 (36.8, 37.7)	37.3 (36.7, 37.9)	37.7 (37.2, 38.2)	37.5 (36.9, 38.0)	<0.001
Lymphocytes (%)	14.8 (9.1, 18.3)	9.9 (5.3, 12.4)	11.9 (6.7, 14.9)	12.1 (6.5, 15.5)	<0.001
Coagulation					
APTT	32.4 (24.9, 35.0)	38.5 (27.0, 45.6)	34.4 (25.5, 38.9)	35.1 (26.0, 40.0)	<0.001
Prothrombin time (sec)	13.4 (11.7, 14.9)	12.9 (11.2, 14.1)	12.9 (11.4, 14.1)	13.0 (11.4, 14.3)	0.016
Medication					
Anticoagulants	192 (76.8%)	277 (96.9%)	385 (97.7%)	854 (91.8%)	<0.001
Fluids	148 (59.2%)	251 (87.8%)	333 (84.5%)	732 (78.7%)	<0.001
Vasopressors	20 (8.0%)	237 (82.9%)	125 (31.7%)	382 (41.1%)	<0.001
Diuretics	66 (26.4%)	160 (55.9%)	124 (31.5%)	350 (37.6%)	<0.001
Corticosteroids	44 (17.6%)	86 (30.1%)	108 (27.4%)	238 (25.6%)	0.002
Other immunosuppressives	1 (0.4%)	4 (1.4%)	8 (2.0%)	13 (1.4%)	0.243
Antibiotics	32 (12.8%)	108 (37.8%)	128 (32.5%)	268 (28.8%)	<0.001
Antimycotics	27 (10.8%)	65 (22.7%)	81 (20.6%)	173 (18.6%)	0.001
Antivirals	12 (4.8%)	32 (11.2%)	26 (6.6%)	70 (7.5%)	0.013
Muscle relaxants	0 (0.0%)	175 (61.2%)	19 (4.8%)	194 (20.9%)	<0.001
Opioids	44 (17.6%)	272 (95.1%)	373 (94.7%)	689 (74.1%)	<0.001
Sedatives	33 (13.2%)	275 (96.2%)	346 (87.8%)	654 (70.3%)	<0.001
